# The use of VivaSight™ single lumen endotracheal tube in morbidly obese patients undergoing laparoscopic sleeve gastrectomy

**DOI:** 10.1186/1471-2253-14-31

**Published:** 2014-05-05

**Authors:** Michal Barak, Ahmad Assalia, Ahmad Mahajna, Bishara Bishara, Alexander Braginski, Yoram Kluger

**Affiliations:** 1 Department of Anesthesiology, Rambam Health Care Campus, and The Bruce Rappaport Faculty of Medicine, Technion-Israel Institute of Technology, Haifa, Israel; 2 Department of General Surgery, Rambam Health Care Campus, and The Bruce Rappaport Faculty of Medicine, Technion-Israel Institute of Technology, 8 Ha’Aliyah Street, Haifa 35254, Israel; 3 Department of Anesthesiology, Baruch Padeh Medical Center, Poriya, Tiberias, Israel

**Keywords:** Equipment, Airway, Anesthetic techniques, Fiber-optic, Ventilation, Mechanical, Morbid obesity, Surgery, Bariatric complications

## Abstract

**Background:**

The population of obese patients is progressively growing and bariatric operations are becoming increasingly common. Morbidly obese patients require special anesthetic care and are often considered to be difficult to ventilate and intubate. The VivaSight™ Single Lumen tube is an endotracheal tube with a camera embedded in its tip. The view from the tip appears continuously on a monitor in the anesthesiologist's vicinity. The aim of this study was to assess the VivaSight™ in comparison with conventional endotracheal tube as an aid in the intubation and surveillance of tube position during surgery of obese patients.

**Methods:**

This is a prospective study of 72 adult obese patients who underwent laparoscopic sleeve gastrectomy. The patients were randomly assigned to be intubated by either the VivaSight™ (40 patients, test group) or a conventional endotracheal tube (32 patients, control group). Data on the patients, the pre-operative airway evaluation, the endotracheal intubation and the post-operative outcome were collected and compared.

**Results:**

The Mallampati scores were significantly higher in the test group than in the control group. Endotracheal intubation took 29 ± 10 and 24 ± 8 seconds using the VivaSight™ and a conventional tube respectively (p = 0.02). Three of the patients in the control group, while none of those in the test group, had soft tissue injury (p < 0.05).

**Conclusion:**

We found the VivaSight™ SL to be helpful in the endotracheal intubation and continuous surveillance of tube position in morbidly obese patients undergoing laparoscopic sleeve gastrectomy.

## Background

The number of obese patients who require bariatric and non-bariatric surgery is progressively increasing [1-3]. The obese patient presents numerous problems and challenges to the surgeon and anesthesiologist [4]. Morbidly obese patients are often difficult to ventilate and difficult to intubate [5,6]. These difficulties may be aggravated in the obese patient by rapidly occurring hypoxemia, due to decreased functional residual capacity and low oxygen reserve [5,6], which, in turn, may lead to significant morbidity and mortality [7]. Several solutions have been suggested and tried for managing the obese patient’s airways and ventilation during anesthesia [8-12].

**Figure 1:**
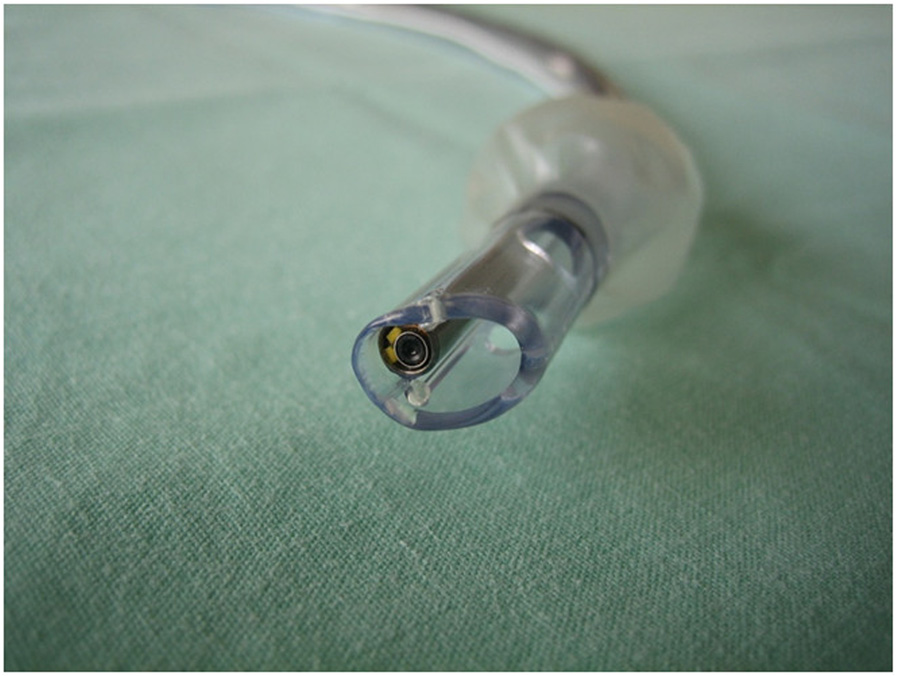
**The VivaSight™ Single Lumen endotracheal tube with a camera embedded in its tip.**

**Figure 2:**
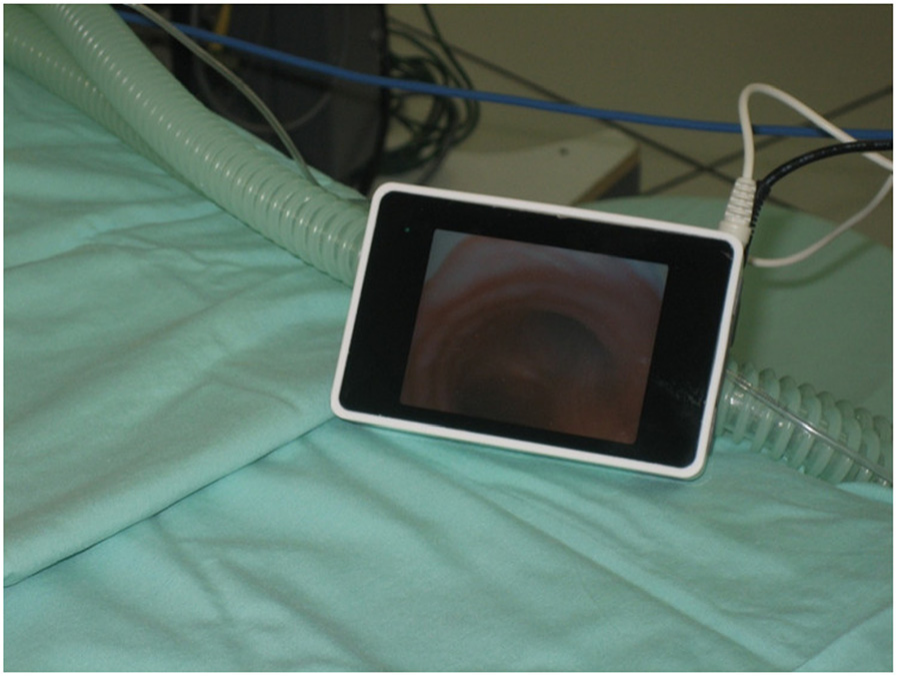
**A clear view of the patient's carina is seen on the screen of the VivaSight™ monitor.**

**Figure 3:**
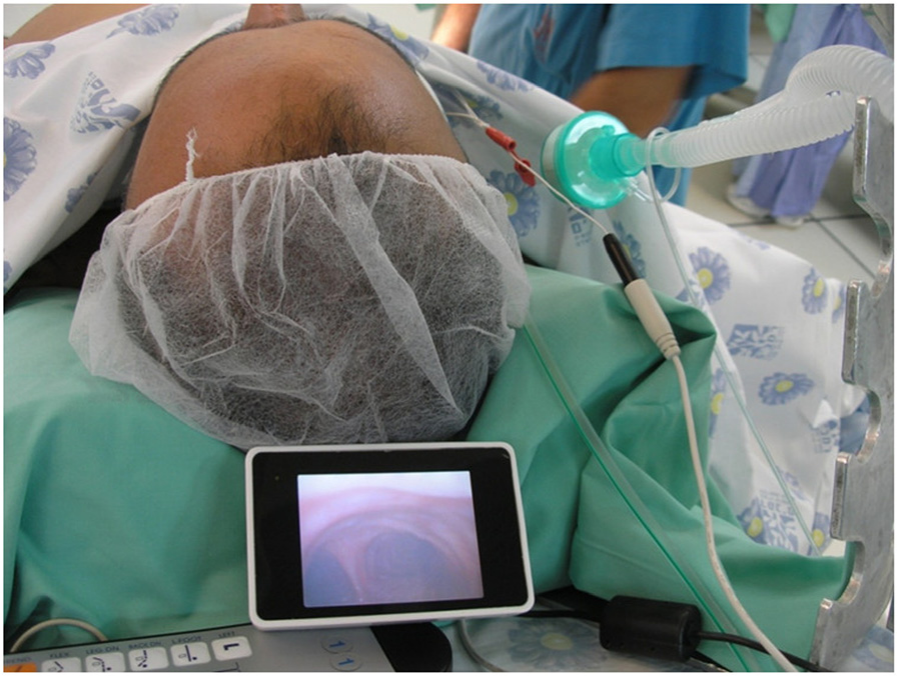
**The patient after endotracheal intubation with the VivaSight™ SL endotracheal tube.** The patient's carina is seen on the screen of the VivaSight™ monitor.

The VivaSight™ Single Lumen (SL) endotracheal tube (ETT) (ETView Ltd., Misgav 20174 Israel) is a single-use ETT with an integrated high-resolution imaging camera embedded in the tube's tip (Figure 1) [13,14]. The external structure and dimensions of the VivaSight™ SL ETT are similar to those of the conventional ETT, and the device is available in sizes 7.0, 7.5 and 8.0 mm. The view from the tube tip appears continuously on a portable monitor (Figures 2, 3) in the anesthesiologist's vicinity. According to the manufacturer, the appliance (a) facilitates fast and efficient intubation, (b) provides visual assurance during intubation, and (c) permits continuous, real-time images of tube position, which can be viewed on a battery- or cable-operated liquid-crystal display (LCD) monitor. The VivaSight™ SL has United States Food and Drug Administration approval and the CE marking.

Laparoscopic sleeve gastrectomy is a bariatric procedure in which a considerable longitudinal part of the stomach is removed and the remaining stomach is shaped into a tube or "sleeve". During laparoscopic sleeve gastrectomy, the ETT position may change due to pneumoperitoneum and the patient's position [15,16]. Confirmation of ETT position by auscultation is especially difficult in obese patients and approaching the patient or the ETT is not always easy during the surgery.

Our hypothesis was that the VivaSight™ SL ETT could be helpful in tracheal intubation and possibly will assist in the surveillance of tube position during laparoscopic sleeve gastrectomy because it facilitates visualization of the patient's airway during and after endotracheal intubation. The study's specific goal was to assess the clinical performance of VivaSight™ SL in comparison with conventional ETT. Primary outcome was intubation time; other features that were tested: direct laryngoscopic view, number of attempts to accomplish intubation and post-operative consequences, such as soft tissue injury. By studying the technical features of its use one can appreciate whether this novel device will add in the management of airway during operations of morbidly obese patients.

## Methods

This was a prospective study of adult patients who underwent elective laparoscopic sleeve gastrectomy in our hospital. The study was approved by the local Ethics Committee (Rambam Health Care Campus Ethics Committee; approval number 0181-09-RMB) and all patients signed an informed consent. Permission to reproduce the images after endotracheal intubation with the VivaSight™ SL ETT in Figures 2 and 3 was obtained from the patient.

The patients were randomly assigned into two groups: a control group of 32 patients which were intubated with a conventional ETT and a test group of 40 patients who were intubated with the VivaSight™ SL ETT. The sample size of the test group was limited by the number of VivaSight™ tubes that were supplied by the appliance's manufacturer. Exclusion criteria were: American Society of Anesthesiologists (ASA) class ≥4, pregnancy, patients who required rapid sequence induction and patients with known tracheal pathology.

All patients in both groups received standard identical care before and during the operation, with the only difference set to be the ETT type used. Each patient was admitted to hospital one day before the scheduled surgery, was examined by a surgeon and an anesthesiologist, who also evaluated their airways. On the day of surgery, each patient was pre-medicated with oral metoclopramide (10 mg) and diazepam (10 mg), which is our hospital's routine pre-medication in all the patients undergoing surgery. In the operating room, the patient was monitored with non-invasive blood pressure, electrocardiogram and pulse-oximeter, and a peripheral intravenous (IV) access was established. Pre-oxygenation was achieved using a face mask which delivered oxygen at 10 liters/min for five minutes before tracheal intubation. General anesthesia in each patient was induced by an intravenous bolus injection of propofol (2–3 mg kg^-1^) and fentanyl (0.001-0.002 mg kg^-1^), followed by vecuronium (0.08 mg kg^-1^). In cases where mask ventilation was difficult, as assessed by the intubating anesthesiologist after the administration of propofol, based on the effort used in ventilation with the bag and on the capnography, vecuronium was not administered and awake fiberoptic intubation was performed; the patient was removed from the study. Anesthesia in all patients was maintained by sevoflurane in a mixture of oxygen and air and a continuous IV infusion of remifentanil. Endotracheal intubation was performed by an experienced anesthesiologist (MB or AB). Each anesthesiologist was trained in the use of the VivaSight™ prior to the study on a manikin with the manufacturer's instructor help. The device was then used on at least a dozen patients, who were not included in the study. Intubation with the tested device or with conventional ETT was performed when hypnosis and muscle relaxation were fully achieved. Intubation was done using a standard 7.5 mm ETT in the control group and a 7.5 mm VivaSight™ SL in the test group. The VivaSight™ ETT was connected to a 3.6"(screen size) monitor and the tube was fixed with adhesive tape and a cotton band when a clear picture of the patient's carina was seen on the monitor's screen (Figure 3). ETT fixation was identical in the control group. Following tube fixation, the patient was placed in a sitting position and prepared for surgery.

During the surgery, the patient's carina was continuously visualized on the monitor's screen by the anesthesiologist and ETT position was recorded every 5 minutes. Tube movement within 1 cm was recorded, as was the need for re-positioning of the tube when the carina was not seen on the monitor's screen. After completion of the surgery and extubation, all patients were transferred to the post-anesthesia care unit (PACU), where they were monitored for one hour. Heart rate, respiratory rate, non-invasive blood pressure and oxygen saturation were continuously monitored in PACU, and recorded once in every 5 minutes by the nurses. A face mask with oxygen (10 L/min) was used in all patients. Oxygen saturation of 90% or lower, respiratory rate above 20/min, breathing exertion, and patient's complaint of breathing difficulty were all reported by the nurse to the attending PACU physician and managed immediately as required.

In each patient, the following variables that were recorded during the pre-operative examination: age, sex, body weight, body mass index (BMI), ASA class, and various parameters of airway assessment: the extent of mouth opening and temporomandibular joint movement, the state of their dentition, the thyro-mental distance, the presence of micrognatia and a short thick neck, and the Mallampati score [17]. The following intra-operative variables were recorded by the attending anesthesiologist: the duration of intubation (from introducing the laryngoscope into the mouth until the tube’s cuff is inflated), direct laryngoscopic view according to the Cormack - Lehane classification [18], the number of intubation attempts until successful intubation was accomplished, and the number of times that the laryngoscope blade needed replacing. The following post-operative consequences were recorded by a PACU nurse, who was blinded to the study group: laryngospasm, stridor, hoarseness, sore throat, tooth damage and soft tissue injury, such as a bleeding lip or injured gums.

### Data analysis

A commercial statistical software package (Statistica 6.0, StatSoft, Tulsa OK, USA) was used to analyze the data. Pearson’s chi square test was used to compare frequencies. Continuous variables were compared using analysis of variance. Whenever the homogeneity of variance was violated according to the results of the Levene’s test, the Mann–Whitney test was used. Data are displayed at mean ± standard deviation (SD) and statistical significance was set at 5%.

## Results

All study patients in the two groups were successfully intubated. No patient was excluded from the study because of difficult mask ventilation or other reason.

Regarding the pre-operative characteristics and airway assessment of the patients, we found that the studied groups were similar in terms of their demographics and ASA physical status (Table 1). The Mallampati scores were significantly higher in the test group than in the control group (p = 0.01).

**Table 1:** The preoperative characteristics and airway assessment of the patients

	**VivaSight™ group**	**Control group**	**P value**
	**(N = 40)**	**(N = 32)**	
Age, years	43.1 (±4.9)	42.5 (±3.2)	NS
Male/Female	14/26	9/23	NS
Weight, kg	128 ± 26	124 ± 27	NS
BMI	44.8 ± 7.5	43 ± 6.8	NS
ASA physical status class I/II/III	17/22/1	12/18/2	NS
**Airway assessment**			
Mouth opening: normal/limited	39/1	32/0	NS
Dentition state: normal/protruding	38/2	31/1	NS
Thyromental distance: normal/short	40/0	31/1	NS
TMJ movement: normal/limited	35/5	31/1	NS
Neck: normal/short	37/3	32	NS
Mallampati score: 1/2/3/4	4/21/14/1	13/14/5/0	P = 0.01

Notes:

Data are displaced at mean ± standard deviation (SD).

NS = not significant.

BMI = body mass index.

ASA = American Society of Anesthesiologists.

TMJ = temporo-mandibular joint.

Endotracheal intubation was significantly longer in the test group. It took 29 ± 10 and 24 ± 8 seconds using the VivaSight™ SL and the conventional endotracheal tube respectively (p = 0.02). However, patients in the control group had significantly more soft tissue damage than those in the test group (p < 0.05) when examined in the PACU. No statistically significant differences in the other study parameters of the two groups were found (Table 2).

**Table 2:** Data regarding endotracheal intubation and post-operative outcome

	**VivaSight™ group**	**Control group**	**P value**
	**(N = 40)**	**(N = 32)**	
**Endotracheal intubation**			
Duration (seconds)	29 ± 10	24 ± 8	P = 0.02
Grades of the direct laryngoscopic view			
1/2/3/4*	14/23/3/0	8/23/1/0	NS
Intubation accomplished at first attempt	39	31	NS
Number of times blade was replaced	0	1	NS
**Post-operative parameters**			
Laryngospasm	0	0	NS
Hoarseness	0	0	NS
Stridor	0	0	NS
Sore throat	2	3	NS
Soft tissue injury	0	3	P < 0.05
Tooth damage	0	0	NS

Notes:

Data are displaced at mean ± standard deviation (SD).

NS = not significant.

*Grades of the direct laryngoscopic views according to the Cormack-Lehane classification [18].

## Discussion

In this study the use of VivaSight™ ETT was compared to the use of conventional ETT in morbidly obese patients. The patients in the test group had a higher Mallampati score distribution than those who were in the control group. Endotracheal intubation using the VivaSight™ ETT took significantly longer than that using a conventional ETT and was less injurious. No significant difference in all other variables was found in the two study groups.

Endotracheal intubation is an essential tool in managing the patient under general anesthesia, yet it may result in airways damage. Injuries, usually of minor degree, of the gums, pharynx and larynx, oedema of the larynx, vocal cord injury, sore throat and more significant complications as tracheal tear, may all be the sequelae of traumatic laryngoscopy and intubation [19,20]. Morbidely obese patients are at higher risk of these complications [5,6,21]. While the technique and device that are used during intubation of the obese patient may affect the outcomes, the superiority of a one method over any other in terms of safety or effectiveness is yet to be determined [21].

One of the limitations of this investigation is the significant difference in the Mallampati score distribution of study groups. Since the patients in the test group had a higher Mallampati score than those in the control group one could assume that they were in higher risk for difficult intubation [17]. This increased risk is partially offset by the similarity in the grades of direct laryngoscopic views in the two groups because this grade is more indicative of intubation difficulty than the Mallampati score. A second limitation of the study is that the ETT position in the control group was not determined with fiberoptic bronchoscopy. Thus we cannot comment about its position and any tube displacement during the surgery. Finally, the size of the study's cohort. It is estimated that difficult direct laryngoscopy occurs in 1.5-8.5% of all general anesthesias and failed intubation occurs in 0.13-0.3% of all general anesthesias [22]. Therefore, in order to obtain significant results in a prospective study whose aim is to evaluate a new device for difficult intubations hundreds of patients are required.

Continuous visual surveillance of ETT position in patients while undergoing bariatric surgery is beneficial for the anesthesiologist and for the patient's safety. While placing the patient in the operating position for laparoscopic sleeve gastrectomy, the ETT may become displaced and its tip might move distally or closer to the carina due to peritoneal inflation [15,16,23,24] and result in ventilation of only one lung. Insertion of an esophageal bougie may also cause tube displacement. Verifying the ETT placement and position in the obese patient using auscultation may be difficult because the breath sounds are quiet and distant. Finally, the working conditions in bariatric laparoscopic surgery, namely a dark or poorly-lit room in which the patient is fully covered and the operating table is elevated, make it very difficult for the anesthesiologist to ensure the ETT is in place or to safely change its location when the ETT becomes displaced. Accordingly, visualization of the carina on the VivaSight™ monitor has a reassuring effect on the attending anesthesiologist, and once the ETT is displaced, it can be re-positioned before hypoxemia ensues.

During difficult intubations, when the laryngoscopic view is poor, the use of VivaSight™ SL may be advantageous. When the laryngoscopic view is good, during direct laryngoscopy, the anesthesiologist can use the device as a standard ETT. When the laryngoscopic view is poor, the anesthesiologist can be assisted by the view on the monitor's screen to direct the ETT into the vocal cords. The anesthesiologist does not have to change equipment or position or repeat the direct laryngoscopy. In addition, no time is lost and no injury to the patient's larynx is caused by a repeated laryngoscopy.

Determining the cost-effectiveness of new equipment is difficult. Picot et al. [25] did an economic evaluation of bariatric surgery and found that the surgical management of obesity (BMI ≥30) was more expensive than non-surgical management in the immediate short-term, but was significantly cost-effective two to 20 years after the surgery. Similar findings were also reported by Sussenbach et al. [26] when they compared the direct and indirect costs of obesity and related co-morbidities. Sussenbach et al. also reported that the cost of the bariatric surgery is high, but the expenses for medications, professional care, and examinations decrease progressively after the surgery [26]. The cost of one disposable VivaSight™ SL ETT is currently about €100 and the monitor, which can be used repeatedly, is €750. Accordingly, their purchase and use increase the cost of the procedure, this should be considered in the light of its possible benefits.

## Conclusions

In this study we found the VivaSight™ SL ETT to be an interesting addition to the armamentarium of airways devices. Intubation with this device took longer and was less injurious than with the conventional ETT in groups of obese patients that differ in their Mallampati scores distribution. Its benefits in the management of the patient with difficult airway are yet to be tested.

## Abbreviations

ETT: Endotracheal tube; ASA: American Society of Anesthesiologists; PACU: Post-anesthesia care unit; BMI: Body mass index; IV: Intravenous.

## Competing interests

The authors declare that they have no competing interests.

## Authors’ contributions

MB conceived the study, accompanied the data acquisition, collected and analyzed the data and drafted the manuscript. AB collected data and helped write the manuscript. AA, AM, BB and YK participated in the design of the study and accompanied the data acquisition. All authors read and approved the final manuscript.

## Pre-publication history

The pre-publication history for this paper can be accessed here:

http://www.biomedcentral.com/1471-2253/14/31/prepub
